# Taxonomic notes on two sibling species of *Metellina* from Asia (Araneae, Tetragnathidae)

**DOI:** 10.3897/zookeys.695.13611

**Published:** 2017-09-04

**Authors:** Recep Sulhi Özkütük, Yuri M. Marusik, Kadir Boğaç Kunt, Mert Elverici

**Affiliations:** 1 Department of Biology, Faculty of Science, Anadolu University, TR-26470, Eskişehir, Turkey; 2 Institute for Biological Problems of the North, Portovaya Street 18, Magadan 685000, Russia; 3 Department of Zoology & Entomology, University of the Free State, Bloemfontein 9300, South Africa; 4 Department of Biological Sciences, Faculty of Arts and Sciences, Middle East Technical University, TR-06800 Ankara, Turkey; 5 Department of Biology, Faculty of Science and Arts, University of Erzincan, TR-24100, Erzincan, Turkey

**Keywords:** Anatolia, Aranei, Metainae, Caucasus, Central Asia, Iran, new record, description

## Abstract

Two sibling species, *Metellina orientalis* (Spassky, 1932) and *M. kirgisica* (Bakhvalov, 1974), occurring in the Middle East and Central Asia are redescribed and their distributions mapped. The previously unknown male of *M. kirgisica* is described for the first time. Stridulatory files on male chelicera of *Metellina* are also documented for the first time. The occurrence of *M. kirgisica* in Azerbaijan and Tajikistan and the presence of *M. orientalis* in Turkey are confirmed.

## Introduction

*Metellina* Chamberlin & Ivie, 1941 is a small genus of Metainae spiders with seven named species occurring in the Holarctic (World Spider Catalog 2017). Two species, *M. curtisi* (McCook, 1894) and *M. mimetoides* Chamberlin & Ivie, 1941, are restricted to the Nearctic and all other species are known from West Palaearctic (from the Iberian Peninsula to Xinjiang). Three species occurring in Europe and both species known from the Nearctic are well studied due to several publications (Levi 1980; Roberts 1995, etc.). Two easternmost Palaearctic species, *M. kirgisica* (Bakhvalov, 1974) and *M. orientalis* (Spassky, 1932), are the least known species of the genus. The former is known by females only and sketchy drawings; although *M. orientalis* is relatively well known, some essential characters of this species are not documented, such as the cymbial spines or stridulatory files on male chelicera, as well as the stiff setae present on legs I and II. Distributions of the two species are not properly known due to past misidentifications. Difficulties were faced in discriminating between these two species during studies of Turkish, Caucasian, and Central Asian spiders; therefore, a comparative study of *M. kirgisica* and *M. orientalis* is provided.

## Materials and methods

Specimens were photographed with a Canon EOS 7D camera attached to an Olympus SZX16 stereomicroscope and Leica DFC295 camera connected to a stereo microscope Leica S8AP0. SEM figures were made with a SEM JEOL JSM-5200 scanning microscope at the Zoological Museum, University of Turku, Finland and with a Zeiss Ultra Plus SEM device at the Anadolu University, Eskişehir. Digital images were montaged using CombineZP image stacking software. The epigyne was cleared in a KOH/water solution until soft tissues were dissolved. Photographs were taken in dishes with cotton or paraffin on the bottom to hold the specimens in position. All measurements are given in mm. Materials studied here are deposited in the Zoological Museum of the Moscow State University (**ZMMU**), Zoological Institute of St-Petersburg (**ZISP**), Zoological Museum, University of Turku (**ZMUT**), and Anadolu University, Zoological Museum (**AUZM**).

## Taxonomy

### Metellina

Taxon classificationAnimaliaAraneaeTetragnathidae

Chamberlin & Ivie, 1941

Metellina Chamberlin & Ivie, 1941: 14; Levi 1980: 32; Álvarez-Padilla and Hormiga 2011: 779.

#### Type species.

*Pachygnatha curtisi* McCook, 1894 from California.

#### Diagnosis.

See Levi (1980) and Álvarez-Padilla and Hormiga (2011).

### Metellina orientalis

Taxon classificationAnimaliaAraneaeTetragnathidae

(Spassky, 1932)

Figs 1–6 , 9–10 , 17–19 , 23–25 , 29 , 30–33 , 34–36 , 39 , 47

Meta orientalis Spassky, 1932: 184, f. 5–8 (♂♀).
Metellina orientalis : Marusik 1985: 139; Marusik 1986: 19, f. 1.1–3 (♂♀); Malek Hosseini et al. 2015: 92, f. 3a–c (♂).

#### Examined specimens.

TURKEY: ***Konya*** Province: 1♂, 2♀ (AUZM), Seydişehir District, Kuyucak Mountain, Kalafat Hill, Ferzene Cave (37°22'49.24"N 31°50'2.10"E), 27.03.2011 (R.S. Özkütük); 5♀ (AUZM), Derebucak District, Çamlık Town, Körükini Cave (37°20'53.94"N 31°37'38.53"E), 10.06.2011 (K.B. Kunt); 1♂, 2♀ (AUZM), Derebucak District, Çamlık Town, Döllüönüini Cave (37°20'20.38"N 31°37'15.12"E), 10.07.2011 (R.S. Özkütük). ***Erzincan*** Province, 2♂, 4♀ (AUZM), Kemaliye District, Kozlupınar Village, Ala Cave (39°13'5.63"N 38°34'20.71"E), 20.03.2015 (M. Elverici) ***Sivas*** Province, 3♂, 1♀ (AUZM), Şarkışla District, Alaman Village, Camızlı Cave (39°35'8.91"N 36°15'12.13"E), 18.04.2016 (K.B. Kunt). ARMENIA: syntype 1♂ (ZISP), Goktscha Lake (=Sevan), Yelenovka, 13–16.08.1931 (M. Karpova). IRAN: ***Kohgiluyeh and Buyer-Ahmad*** Province, 1♂ 1♀ (ZMUT), Nevel Cave, 28.08.2011 (M.J. Malek Hosseini).

**Figures 1–6.**
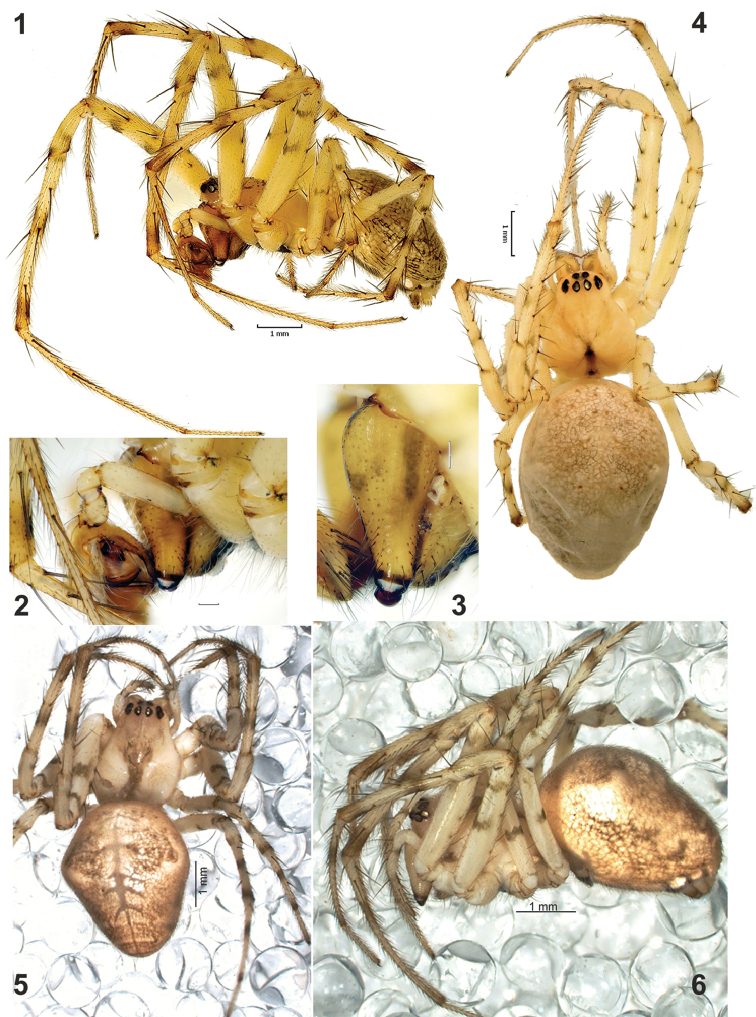
Somatic characters of *Metellina orientalis*. **1** male habitus, lateral **2–3** anterior part of prosoma showing chelicera with stridulating ridges **4–5** female habitus, dorsal; **6** female habitus, lateral. Scale bars 1 mm (**1, 4, 5, 6**); 0.2 mm (**2**).

**Figures 7–10.**
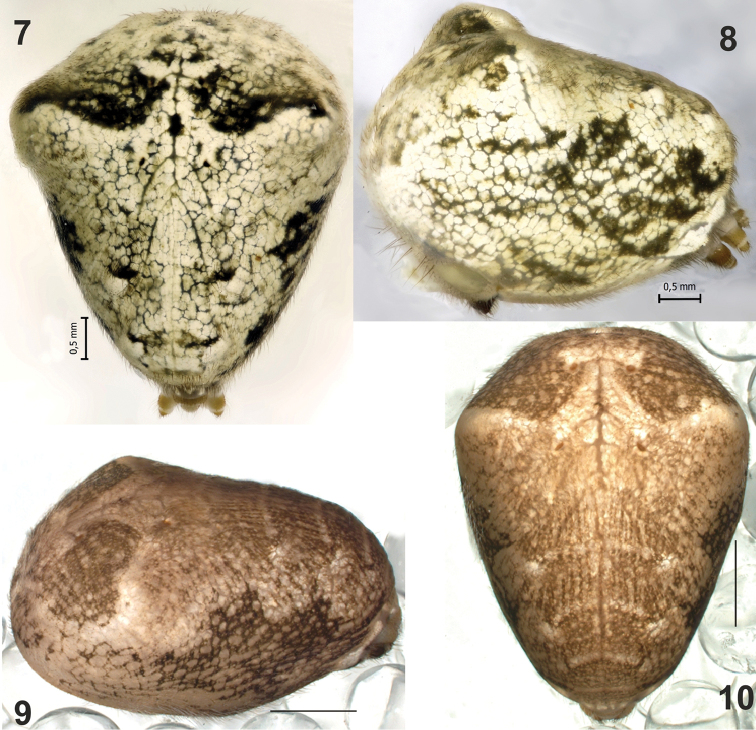
Abdomen of *Metellina kirgisica* (**7–8**) from Azerbaijan and *M. orientalis* (**9–10**) from Erzincan Province of Turkey. **7, 10** dorsal **8–9** lateral. Scalebars 0.5 mm (**7, 8**); 1 mm (**9, 10**).

#### Diagnosis.

**Figures 11–16.**
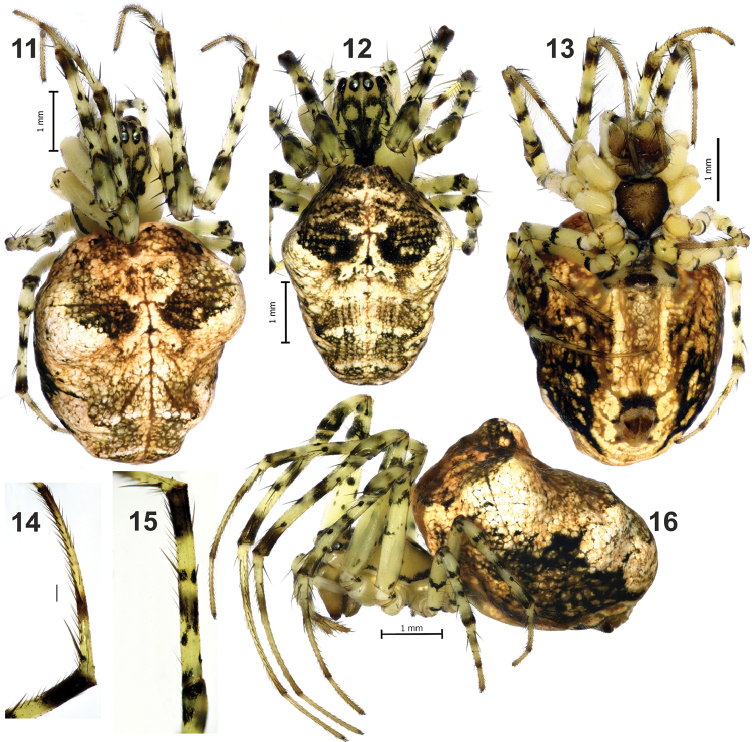
Somatic characters of *Metellina kirgisica* female from Tajikistan. **11–12** habitus, dorsal, showing differences in size and colour pattern **13, 16** habitus, ventral and lateral **14** metatarsus I prolateral, showing row of stiff setae **15** tibia I, prolateral. Scale bars 1 mm (**11, 12, 13, 16**); 0.2 mm (**14**).

**Figures 17–22.**
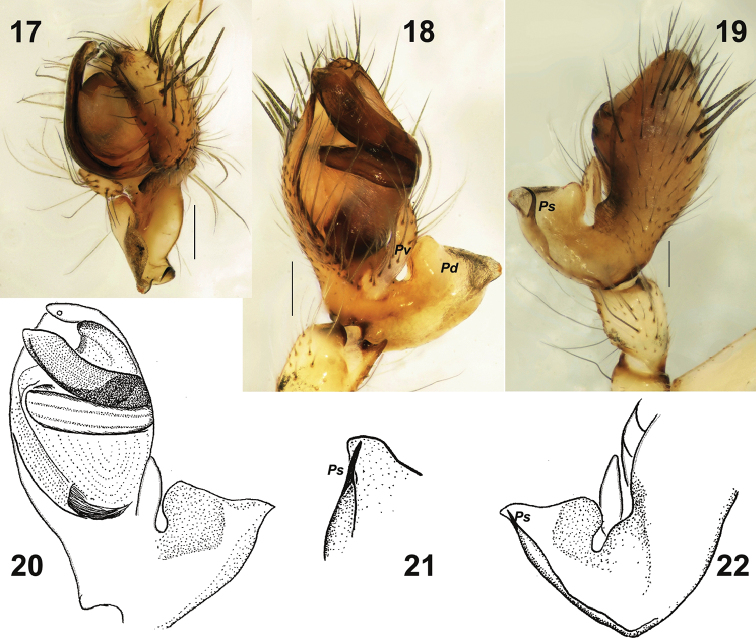
Male palp of *Metellina orientalis* (**17–19**) from Azerbaijan and *M. kirgisica* (**20–22**). **17** from above **18, 20** ventral **19, 22** dorsal **21** tip of paracymbium with spur. *Pd* dorso-retrolateral arm *Ps* spur like process *Pv* finger-like ventral arm. Scale bars 0.2 mm (**17, 18, 19**).

**Figures 23–29.**
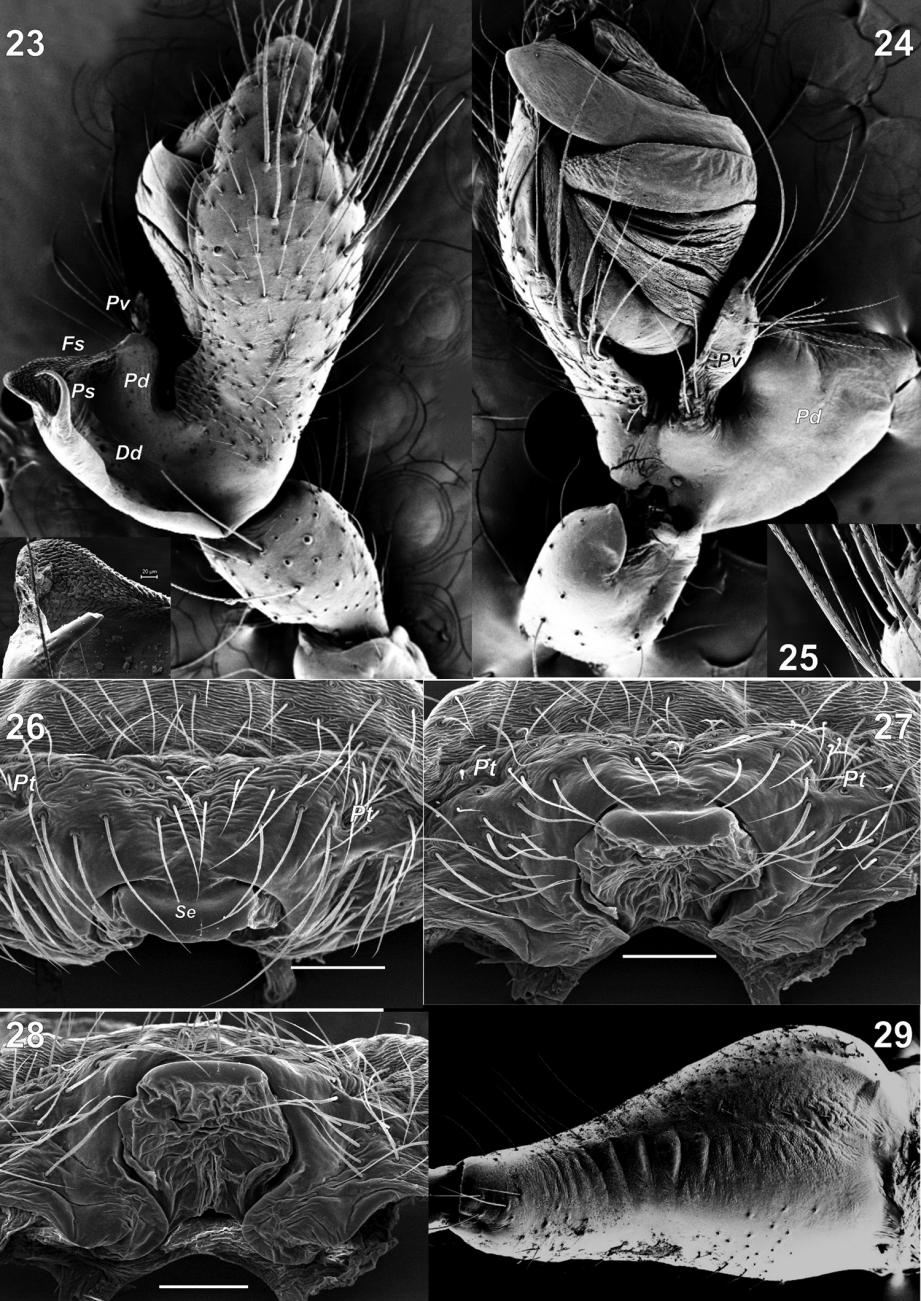
Copulatory organs and chelicera of *Metellina orientalis* (**23–25, 29**) from Konya Province of Turkey and *M. kirgisica* (**26–28**). **23–24** male palp, dorsal and ventral **25** cymbial setae **26–28** epigyne, ventral, ventro-caudal and caudal **29** male chelicera, lateral. Abbreviations: *Dd* deep depression *Fs* fine spines *Pd* dorso-retrolateral arm *Ps* spur like process *Pt* lateral pits *Pv* finger-like ventral arm *Se* sclerotised outgrowth. Scale bars 0.2 mm (**26, 27, 28**).

Males of *M. orientalis* can be easily distinguished from all congeners, except for *M. kirgisica*, by having strong cymbial spines (Figs 17–19, 23, 25) lacking in other species. The two sibling species can be distinguished by the shape of paracymbial spur (*Ps*), which are rounded and claw-like in *M. orientalis* (Figs 19, 23) and spine-like in *M. kirgisica* (Figs 21–22). Females of *M. orientalis* are also very similar to those of *M. kirgisica* by having three pairs of abdominal humps (can be almost indistinct in some specimens) and a very similar epigyne. The epigyne in *M. orientalis* has a larger and wider median plate (cf. Fig. 36, 39–40, 42) and a thinner “septum” (1/3 of median plate width vs. 1/2). Females can be easily distinguished by carapace pattern, poorly developed in *M. orientalis* (Fig. 4–5) and very complex in *M. kirgisica* (Figs 11–12).

#### Description.

Measurements (♂/♀): total length 6.48/6.60; carapace 2.88/2.70 long, 2.24/2.04 wide; chelicerae 1.20/1.20 long; abdomen 3.60/3.90 long, 2.40/3.12 wide.

Female carapace with weak pattern (Fig. 5) or almost lacking any pattern (Fig. 4). Legs with numerous spines, stronger in males than in females. Legs light-coloured; femora, tibia and metatarsi of all legs with two dark rings (Figs 1, 4–6). Tibia-metatarsi of legs I and II with prolateral row of stiff, inflexible setae forming kind of catching basket. For leg measurements see Table 1.

**Table 1.** Leg measurements of *Metellina orientalis*.

♂/♀	Fe	Pa	Ti	Mt	Ta	Total
I	3.50/3.60	1.44/1.40	4.00/3.60	4.40/4.00	1.60/1.50	14.98/14.14
II	3.10/3.00	1.20/1.14	3.00/2.22	3.40/3.06	1.20/1.00	11.90/10.42
III	2.00/2.10	0.75/0.84	1.50/2.04	1.80/1.62	0.65/0.78	6.70/7.38
IV	2.88/2.94	0.78/0.84	2.10/2.28	2.40/2.40	0.90/0.90	9.06/9.36

Abdomen with three pairs of humps, almost indistinct in males. Anterior pair large and distinct in all females examined, two posterior pairs much smaller and can be indistinct. Pattern not distinct, in contrast to sibling species; venter with wide dark median band.

**Figures 30–33.**
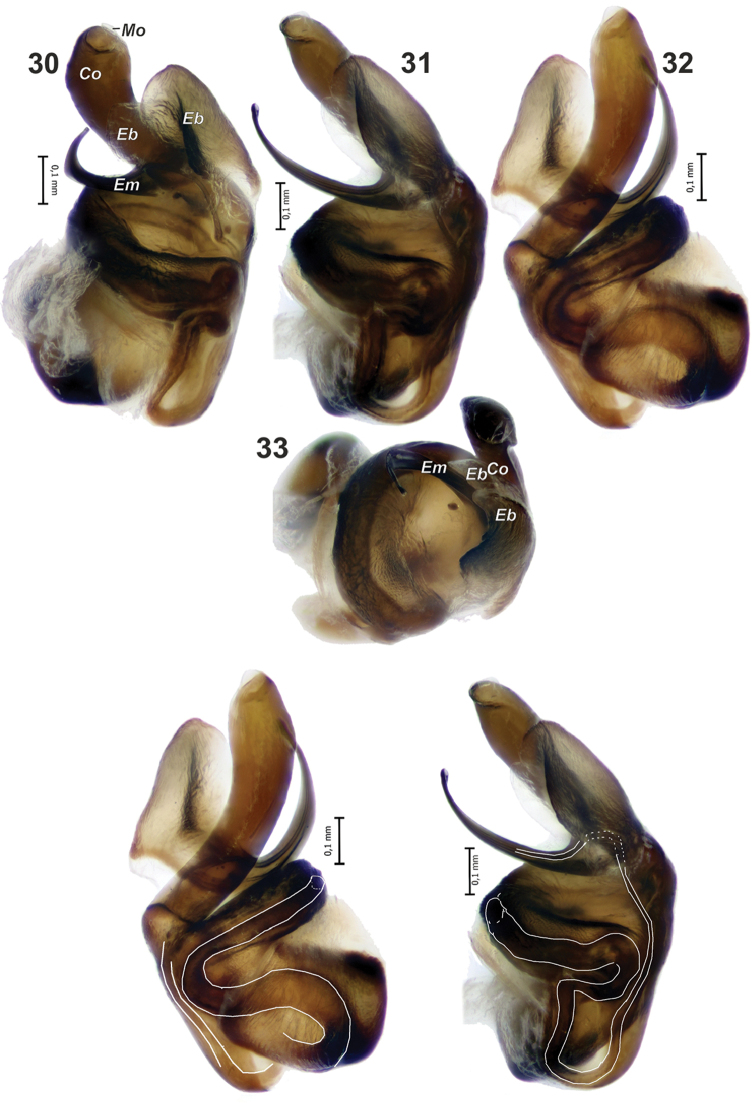
Bulb of *Metellina orientalis*. **33** from above. Abbreviations: *Co* conductor *Eb* embolar base *Em* embolus *Mo* membranous outgrowth. Scale bars 0.1 mm.

Male chelicera with five strong stridulating ridges and some smaller ones above and below. Male palp as in Figs 17–19, 23, 24, 30–33. Cymbium with more than a dozen strong macrosetae in distal half. Paracymbium with finger-like ventral arm (*Pv*) covered with setae and a large extending dorso-retrolateral arm (*Pd*). Dorso-retrolateral arm gradually widens, its width subequal to width of cymbium. Dorso-distal part of the arm with deep depression dorsally (*Dd*), spur like process (*Ps*) and several rows of fine spines (*Fs*) clearly visible with SEM, but indistinct with light microscopy. Tegulum thin, as wide as conductor, transverse. Conductor (*Co*) long, with parallel margins, tip abrupt, with small membranous outgrowth (*Mo*), conductor entirely hides embolus in ventral view; embolus (*Em*) with large base (*Eb*) formed by two lobes; embolus gradually tapering, with widened tip.

**Figures 34–42.**
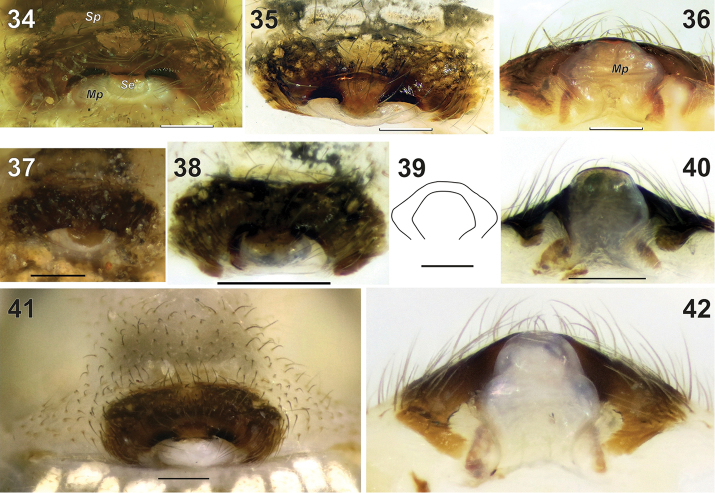
Epigynes of *Metellina orientalis* (**34–36, 39**) and *M. kirgisica* (**37–42**). **34–35, 37–38, 41** ventral **36, 40, 42** caudal **39** outline of median plate of two species, showing differences in size and proportions **34–36** from Konya Province of Turkey **37–40** from Tajikistan **41–42** from Azerbaijan. Abbreviations: *Sp* sclerotized plate *Se* sclerotised outgrowth *Mp* median plate. Scale bars 0.2 mm

Epigyne as in Figs 34–36, 39; simple, heavily sclerotized plate more than twice as wide than long, without any outgrowths; median plate (*Mp*) with septum-like sclerotised outgrowth (*Se*) three times thinner than width of median plate; median plate hexagonal, weakly sclerotized, wider than long. Anterior from epigynal plate with pair of transversal sclerotized plates (*Sp*, Fig. 34).

#### Distribution and notes.

World Spider Catalog (2017) indicates distribution of the species as “Central Asia, Iran” although it was described from Armenia, located in the Caucasus and neighbouring with Turkey, which belong to the Middle East. Mikhailov’s catalogue (Mikhailov 2013) indicates the distribution of *M. orientalis* in the former USSR as Armenia, Kazakhstan, and Turkmenistan: *Metellina orientalis* was reported from Kazakhstan (Almaty) and Turkmenistan (Akhal-Teke) by Spassky (1952) but the two records of the species from Central Asia undoubtedly refer to the sibling species *M. kirgisica*, previously reported from Kyrgyzstan, Uzbekistan, Turkmenistan (Mikhailov 2013), and northwestern China, Xinjiang (Marusik et al. 2007).

This species was reported from Turkey for the first time by Karol (1967). She referred to Spassky (1932) and Charitonov (1936), although none of these publications deal with spiders of Turkey. Spassky (1932) described it from Armenia, and Charitonov (1936) just listed the species in his catalogue. Several surveys and checklists of Turkish spiders listed this species as occurring in Turkey with reference to Karol’s (1967) publications (Bayram 2002; Topçu et al. 2005; Bayram et al. 2017). Now we are able to confirm the presence of *M. orientalis* in Turkey.

### Metellina kirgisica

Taxon classificationAnimaliaAraneaeTetragnathidae

(Bakhvalov, 1974)

Figs 7–8 , 11–15 , 20–22 , 26–28 , 37–42 , 43–46 , 47

Meta orientalis : Spassky, 1952: 1977–198 (misidentification).
Meta kirgisicus Bakhvalov, 1974: 101, f. 6–7 (♀).
Meta kirgisica Bakhvalov, 1982: 136, f. 1 (♀); Bakhvalov 1983: 86, f. 1 (♀).
Metellina kirgisica : Marusik, 1989: 44; Marusik et al. 2007: 271, f. 31, 52 (♀).

#### Material examined.

AZERBAIJAN: ***Lenkoran*** Dist.: 1♀ (ZMMU), env. of Aurora Vill., 38°40'N, 48°52'E, 23–28.04.2001 (Y.M. Marusik). KYRGYZSTAN: 1♂ (lost), Kirgizian Mt. Range, Ala-Archa River, ca. 42.645°N 74.480°E, 8.05.1983 (S.V. Ovtchinnikov); 1♀ (lost), Chatkal Mt. Range, Sary-Chelek Reserve, Karangitun Gorge, ca. 41°40'N, 71°56'E, 3.05.1983 (S.L. Zonstein). TAJIKISTAN: ***Khatlon*** Area: 4♀ (ZMMU), Vose Distr., Khodzha-Mumin Mt., 37°45.941'N, 69°38.665'E, 474 m, 25.04.2015 (Y.M. Marusik); 1♀(ZMMU), Khovaling Distr., Darai-Mukhtor, env. of ”Vose Museum”, 38°23.572'N, 69°57.910'E, 1579 m, 28.04.2015 (Y.M. Marusik); 2♀ (ZMMU) Hissar Mt. Range, Ramit Reserve, 38°44.605'N, 69°18.486'E, 1324 m, 1.05.2015 (Y.M. Marusik); 1♀ (ZMMU), environs of Dushanbe, Hissar Mt. Ridge, 38^th^ km of Varzob Hwy, Takob Gorge, env. of Dehmalik Vill, 38°50.829'N, 68°54.637'E, 805 m, 8.05.2015 (Y.M. Marusik & M. Saidov). CHINA, ***Xinjiang*** Province 1♀ (ZMUT), 70 km southwest of Urumqi, Nantaizi, 43.399°N to 43.438°N, 87.214°E to 87.262°E, 1800–2100 m, 3.05.-28.06.2004 (N.R. Fritzén).

#### Note.

Bakhvalov (1974) described *Meta kirgisicus* only in a key to the orb-weaving spiders of Kyrgyzstan. The type material was not mentioned. In the subsequent paper Bakhvalov (1982) described the same species as *Meta kirgisica* on the basis of the holotype female and several paratypes. This description was supplemented with new figures. A year later this species was described again based on the same material and figures (Bakhvalov, 1983). Bakhvalov (1982, 1983) indicated that types will be deposited in the Laboratory of Entomology of the Institute of Biology of Kirgizian Academy of Sciences. According to Sergei L. Zonstein (pers. comm.), who was working in that laboratory, Bakhvalov never deposited type specimens in the Laboratory of Entomology. After the death of Bakhvalov his private collection was taken by son, and its fate is unknown.

#### Diagnosis.

Females of *M. kirgisica* can be distinguished from sibling *M. orientalis* by smaller size (carapace 2.0–2.1 long *vs.* 2.7), more developed pattern of carapace (cf. Figs 4–6 and 11–12, 16), and proportions of the median plate of epigyne (as long as wide *vs.* wider than long). Males of the two species can be distinguished by the shape of paracymbial spur (*Ps*), straight and spine-like in *M. kirgisica* and bent claw-like in *M. orientalis* (cf. Figs 19 and 21–22).

#### Description.

Male. Measurements (male unavailable, specimen lost, palp was illustrated in 80th by YM). Female: total length 4.5–5.5; carapace 2.0–2.1 long, 1.5–1.7 wide. Carapace yellow with complex dark pattern and distinct marginal dark stripe (Figs 11–12, 16). Legs yellowish with dark annulation and dark spots around base of each spine; femora, tibia and metatarsus of legs with two dark rings, rings of femora thick and thin on tibia and metatarsi; coxae IV with blackish dot (Fig. 13); distal half of tibia and metatarsi I and II with row of stiff setae forming a kind of catching basket (Figs 14–15). For leg measurements see Table 2.

**Table 2.** Leg measurements of *Metellina kirgisica*.

♀	Fe	Pt	Ti	Mt	Ta	Total
I	2.70	1.10	2.85	2.60	1.25	10.50
II	2.20	0.90	1.80	2.10	0.95	7.95
III	1.50	0.65	0.90	1.10	0.65	3.80
IV	2.15	0.70	1.40	1.55	0.75	6.50

Abdomen with three pairs of humps, anterior the largest, two posterior humps less distinct. Abdomen with distinct pattern as shown on Figs 11–13, 16; venter with wide light band.

Palp as in Figs 20–22; spination of cymbium not documented; paracymbium with weakly sclerotised ventral arm and large and broad dorsal arm; dorsal arm with spine-like spur (*Ps*).

Epigyne as in Figs 26–28, 37–46; almost instinct transversal sclerotised plate (*Sp*, Fig. 44) in front of epigynal plate, epigynal plate twice as wide as long, heavily sclerotised with pair of small lateral pits (*Pt*); median plate (*Mp*) weakly sclerotised except kind of septum (*Se*), septum twice as thin as median plate; median plate longer than wide; receptacles touching each other consisting of two lobes (Fig. 46).

**Figures 43–46.**
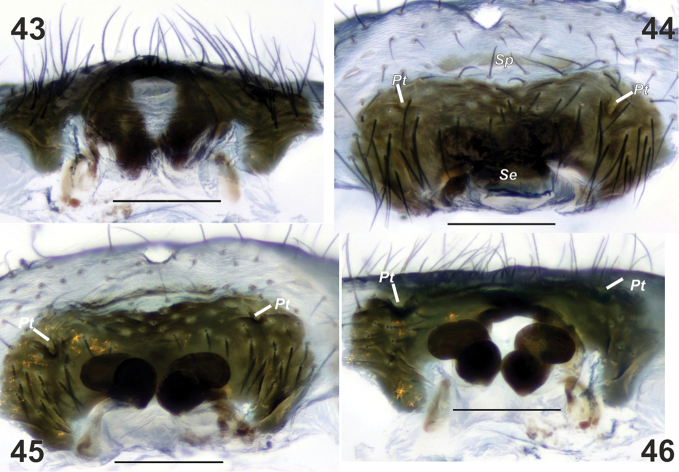
Macerated epigyne of *Metellina kirgisica*. **43** caudal **44** ventral **45** anterior **46** dorsal. ***Pt*** lateral pits ***Se*** sclerotised outgrowth ***Sp*** sclerotized plate. Scale bars 0.2 mm

#### Distribution.

**Figure 47.**
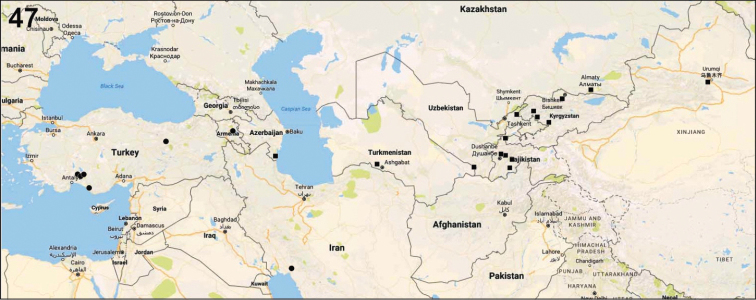
Distribution records of *Metellina orientalis* (circle) and *M. kirgisica* (square).

The species was previously known from Kyrgyzstan, Uzbekistan, Turkmenistan (Marusik 1989; Mikhailov 2013), and Xinjiang, China (Marusik et al. 2007). A search for literature records reveals that it was reported also from southeastern Kazakhstan (sub. *M. orientalis*: Spassky 1952). New material studied in this work reveals its occurrence in Tajikistan and eastern Azerbaijan (Fig. 47).

## Supplementary Material

XML Treatment for Metellina

XML Treatment for Metellina orientalis

XML Treatment for Metellina kirgisica
